# Role of Fibrinolysis in the Management of Patients with COVID-19 and Thromboembolic Complications: A Review

**DOI:** 10.3390/jcdd9100356

**Published:** 2022-10-17

**Authors:** Patrycja Zając, Karol Kaziród-Wolski, Izabela Oleś, Janusz Sielski, Zbigniew Siudak

**Affiliations:** 1 The Reumatology Department, Province Hospital in Konskie, Poland ul. Gimnazjalna 41B, 26-200 Końskie, Poland; 2 Collegium Medicum, Jan Kochanowski University in Kielce, al. IX Wieków Kielc 19A, 25-369 Kielce, Poland

**Keywords:** fibrinolysis, COVID-19, venosus thromboembolism, pulmonary embolism

## Abstract

An impaired fibrinolytic process has been demonstrated in patients infected with SARS-CoV-2, including those in severe or critical condition. Disruption of fibrinolysis leads to fibrin deposition, which exacerbates inflammation and fibrosis and damages the pulmonary surfactant. Numerous authors point out the different course of coagulopathy in patients with COVID-19. It is reported that they may have a state of secondary hyperfibrinolysis, which may explain, at least in part, the increased incidence of venous thromboembolism, even among those patients already receiving appropriate anticoagulant treatment. This raises the question of whether current guidelines for the prevention and treatment of embolic–thrombotic complications, among patients with severe COVID-19, are sufficient. Some studies show evidence of clinical improvement in patients who have received fibrinolytic therapy, beyond the current indications for its implementation. However, when considering the inclusion of systemic fibrinolytic therapy, the benefits of such treatment should always be weighed over the risk of adverse effects. Thromboelastography and rotational thromboelastometry can be helpful in making such decisions. The purpose of this study was to review the current knowledge regarding fibrinolysis and its role in the treatment of patients with severe COVID-19, including those with thromboembolic complications.

## 1. Fibrinogen

Fibrinogen, produced by the liver, is an important protein involved in the final step of blood clotting. After conversion to fibrin via a reaction with thrombin, fibrinogen forms an insoluble clot to prevent bleeding. Fibrinogen is also an acute-phase protein. Its elevated levels are found in patients with infections, injuries, cancer, stress-related disorders and increased cardiovascular disease (CVD) mortality [1,2]. During Coronavirus Disease 2019 (COVID-19) infection, a mean fibrinogen concentration around the upper limit of normal was observed, while patients who died showed a sudden reduction in fibrinogen before death [3,4]. Li et al. [5] studied hematological covariates in 1449 hospitalized patients with COVID-19. At baseline, patients who died had higher fibrinogen levels than survivors (median, 4.3 g/L (interquartile range [IQR], 3.2–5.2 g/L) vs. 3.6 g/L (IQR, 2.9–4.5 g/L)), but they had lower minimum fibrinogen levels (2.6 g/L (IQR, 1.7–3.9 g/L)) vs. 3.2 g/L (IQR, 2.6–3.9 g/L) [5]. The authors concluded that the dynamic changes in fibrinogen levels are correlated with the risk of death in patients with COVID-19. While fibrinogen testing is not routinely performed in all COVID-19 patients on admission to the hospital [6], this parameter might be useful in the early assessment of coagulation in this population.

## 2. Fibrinolysis

Fibrinolysis is a normal body process that is an integral part of the hemostatic system. The activation of the fibrinolytic cascade results in fibrin degradation. Fibrinolysis is regulated by numerous enzymes, with plasmin being the key enzyme involved in the fibrinolytic system. Plasmin is responsible for the lysis of the fibrin clot, thus producing fibrin degradation products. Thrombin is also an important enzyme in the regulation of the coagulation system. Endogenous fibrinolysis is also strongly linked to thrombin, an enzyme responsible for significant resistance to fibrinolysis. High thrombin levels act as a potent platelet activator and facilitate the formation of stable clots by converting fibrinogen into fibrin, which binds platelets together. Moreover, thrombin inhibits endogenous fibrinolysis by activating thrombin-activatable fibrinolysis inhibitor, which prevents the binding and activation of plasminogen into plasmin [7]. Finally, it indirectly inhibits endogenous fibrinolysis by releasing plasminogen activator inhibitor-1 (PAI-1) from platelets, a principal inhibitor of tissue plasminogen activator (tPA) [7].

## 3. Fibrinolysis and COVID-19

An impaired fibrinolytic process has been demonstrated in patients infected with SARS-CoV-2 with interstitial lung disease, including critically ill patients [8,9,10]. Hypercoagulation in these patients is a complex and multifactorial process. SARS-CoV-2 enters the cells via the angiotensin-converting enzyme 2 (ACE2) receptor. It is considered that with SARS-CoV-2 virus attachment, excess angiotensin increases the expression of PAI-1, which inhibits fibrinolysis on endothelial cells [3,11]. Patients with interstitial lung disease were also reported to have elevated levels of thrombin-activatable fibrinolysis inhibitor and protein C inhibitor (plasminogen activator inhibitor-3) [9]. SARS-CoV-2 infection leads to acute inflammation, which leads to an increase in bradykinin and tPA. However, increased tPA levels are not sufficient to counterbalance the high levels of PAI-1, which likely explains the impaired fibrinolysis and fibrin accumulation in the lung alveoli [12]. Fibrin deposits enhance inflammation and fibrosis, and lead to lung surfactant damage [9].

## 4. Role of Plasmin in COVID-19

Plasmin is the major fibrinolytic protease. In their review article, Hong-Long et al. [13] summarized the current evidence for the presence of elevated plasmin levels in COVID-19 patients with hypertension, diabetes, coronary artery disease, cerebrovascular disorders, chronic obstructive pulmonary disease and kidney dysfunction. Plasmin, along with other proteases, may contribute to increased SARS-CoV-2 infectivity, and antiproteases targeting the plasmin(ogen) system may be a promising therapeutic option in patients with COVID-19 [13]. There is also an increasing body of evidence that systemic fibrinolysis may be an effective therapeutic approach in critically ill patients with COVID-19 [12,13,14]. Medcalf et al. [15] described the so-called plasmin paradox in patients with COVID-19, wherein plasmin formation can be either harmful or beneficial, although not at the same time. The authors suggested that an appropriate therapeutic approach aimed either at promoting or inhibiting fibrinolytic activity depends on the severity of infection. In patients with mild or moderate infection, suppression of fibrinolysis may reduce the amount of the virus and improve the immune response. On the other hand, activation of the fibrinolytic system may be protective in severe cases [15]. These conclusions may have important implications for management decisions in patients with COVID-19.

## 5. COVID-19-Associated Coagulopathy and Sepsis-Induced Coagulopathy/Disseminated Intravascular Coagulation

Many authors note the different course of coagulopathy in patients with COVID-19 (CAC, COVID-19-associated coagulopathy) compared to coagulopathy during bacterial sepsis (SIC, sepsis-induced coagulopathy)/disseminated intravascular coagulation (DIC) syndrome. In hospitalized patients with COVID-19, an important coagulation disorder is high D-dimer levels, which are reported to be a negative prognostic factor associated with both increased disease severity and high mortality rates [16]. Although the increase in dimers was initially misinterpreted as a marker of increased fibrinolysis [16], it is now indicated that the essence of CAC is massive fibrin formation, not degradation [17]. The causes of this coagulopathy have not been fully explained; it is thought that a dysregulation of the immune response by inflammatory cytokines, lymphocyte cell death, hypoxia and endothelial damage may be responsible for the abnormal coagulation process in patients with COVID-19 [17]. The hyperinflammatory response, which occurs in most severe cases, can directly and indirectly activate the coagulation cascade; in addition, endothelial cells damaged by the cytokine storm, such as macrophages and monocytes, release TF, activating the extrinsic coagulation pathway and enhancing the coagulation process [16].

Critically ill patients with COVID-19, hospitalized in intensive care units, are at risk for secondary infections and their complications. Ripa et al. studied the incidence of secondary bacterial infections or possible lower respiratory tract infections occurring 48 h after hospital admission until death or discharge. The overall cumulative incidence after 28 days was 16.4% (95% CI, 12.4–21.0%), so the incidence of secondary bacterial infections can be considered high [18]. Bacterial infection may be the starting point of sepsis, with an overlapping risk of progression of coagulopathy to DIC in hospitalized patients with COVID-19. Early detection of infection, prior to decompensation in patients, and the implementation of appropriate treatment therefore appear to be important in its treatment.

Mechanisms affecting SIC and DIC include the coagulation system, platelets, inflammatory cells and damage to the vascular endothelium. The vascular endothelium plays a very important role in this type of disorder, as, under physiological conditions, it releases nitric oxide and prostacyclin, thus contributing to the anticoagulant effect, while, during septic conditions, it promotes prothrombotic effects by expressing the tissue factor and releasing the von Willebrand factor [19]. Endothelial cells, through the production of tissue-type plasminogen activator and PAI-1, regulate fibrinolysis, which is suppressed during SIC/DIC, resulting in the formation of fibrin clots in the tissue microcirculation [19]. In patients with sepsis, coagulopathy can progress from the compensatory phase to the uncompensated phase of DIC, in which thrombosis and bleeding coexist as a consequence of coagulation activation and coagulation factor consumption. Laboratory criteria for diagnosing SIC include thrombocytopenia and a prolonged prothrombin time, among others. The development of thrombocytopenia is often key to the diagnosis of this type of coagulopathy, but this condition should be differentiated from possible other causes of decreased platelet levels in critically ill patients.

To summarize the differences in laboratory deviations in SIC/DIC and CAC, the first aspect is the increase in D-dimers and fibrinogen, which is characteristic of CAC. Of further note are the relatively small deviations in other coagulation biomarkers, including a relatively normal PT level and a slight decrease in platelets, while these two deviations are more common in SIC/DIC [20]. However, platelets in CAC, even if they remain normal, are overactive and have increased interactions with leukocytes, and their hyperactivity leads to the increased release of α-granules and CK, which contributes to inflammation and thrombosis [16]. In anticoagulation protein deviations, the antithrombin level in SIC/DIC decreases significantly, which can be associated, among other aspects, with increased vascular permeability [19]; such a phenomenon has not been observed in CAC, where antithrombin levels either remain normal or remain at the lower end of the reference value range. The main differences regarding the laboratory parameters of the coagulation system in CAC and SIC/DIC are shown in Table 1.

## 6. Venous Thromboembolism and Pulmonary Embolism in COVID-19

Already in the early phase of the pandemic, a very high incidence of venous thromboembolism (VTE) incidents was noted in patients with COVID-19, and thrombotic symptoms involved not only the venous side, but also the arterial side. Moreover, in addition to macrovascular complications, COVID-19 appears to be associated with microvascular thrombosis [16]. Enhanced levels of D-dimers and fibrin or fibrinogen degradation products indicate that massive fibrin formation is the essence of coagulopathy in COVID-19 [17]. The fibrinolytic system is closely linked to D-dimers, a degradation product of fibrin. In COVID-19 patients with suspicion of VTE and pulmonary embolism (PE), D-dimer testing plays a key role. Numerous studies have linked elevated D-dimer levels with worse prognosis [21]. Moreover, admission D-dimer testing showing a more than four-fold increase in D-dimer levels may be a useful prognostic marker of in-hospital mortality in patients with COVID-19 [23].

**Figure 1:**
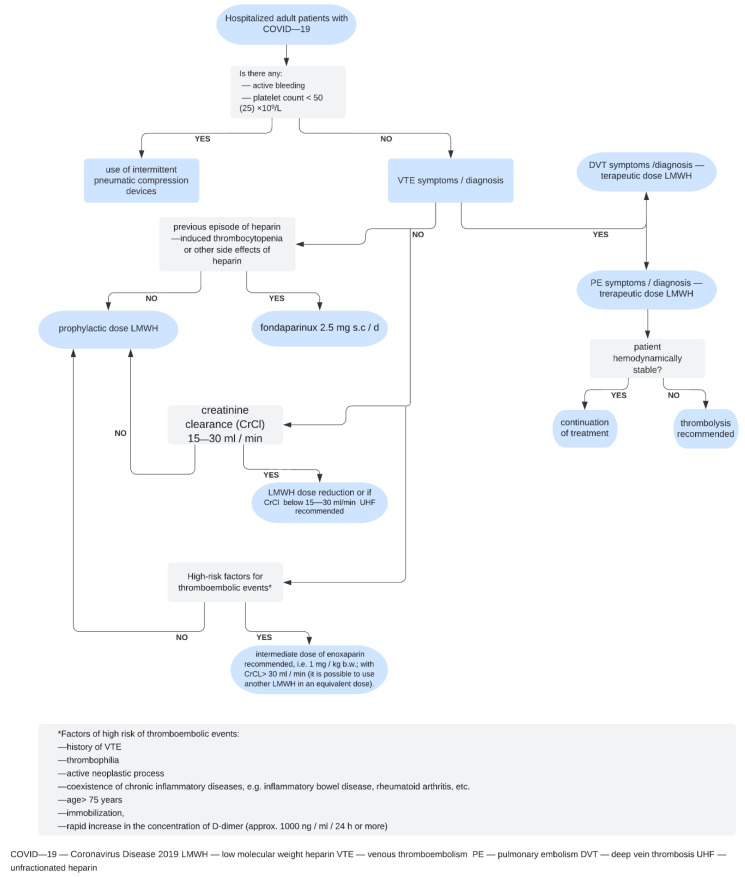
An exemplary algorithm for thromboprophylaxis and anticoagulant treatment in adult patients hospitalized due to COVID-19 based on current expert recommendations.

Manzoor et al. [12] suggested that, similar to fibrinogen, a reduction in D-dimer levels correlating with clinical deterioration may indicate the suppression of the fibrinolytic system. However, Tang et al. [4] reported moderately or markedly elevated levels of D-dimer and fibrin degradation products in all patients who died from novel coronavirus pneumonia, which suggests coagulation activation and secondary hyperfibrinolysis in these patients [4]. Bouck et al. [24] assessed differences in plasma coagulation and fibrinolytic potential between patients with COVID-19, critically ill patients with sepsis and healthy donors. Patients with COVID-19 and sepsis had higher levels of fibrinogen, D-dimer and plasmin–antiplasmin complexes than healthy donors. Moreover, COVID-19 patients had higher thrombin generation potential despite anticoagulant prophylaxis, as well as higher endogenous plasmin potential. Moreover, the lag times to plasmin, thrombin and fibrin formation were prolonged with the increasing severity of COVID-19 [24]. Coagulation disorders with systemic microthrombi and increased incidence of VTE were also confirmed by autopsy studies in patients with COVID-19 [12,25,26]. In a systematic review and meta-analysis, Suh et al. [27] assessed the incidence of PE and DVT in patients with COVID-19. The pooled incidence rates of PE and DVT were 16.5% (95% confidence interval (CI), 11.6–22.9) and 14.8% (95% CI, 8.5–24.5), respectively, and exceeded 20% among patients admitted to the intensive care unit [27]. The treatment and prevention of VTE, including PE, in patients with COVID-19 mostly follows the guidelines for patients without COVID-19. According to the Anticoagulation Forum [28] guidelines, standard-dose pharmacologic VTE prophylaxis is recommended in hospitalized patients with COVID-19. In critically ill patients, increased doses of VTE prophylaxis are recommended. For the treatment of confirmed or suspected VTE in patients with COVID-19, the authors suggest using low-molecular-weight heparin over unfractionated heparin whenever possible. In patients with COVID-19 suspected to have a hospital-associated VTE event, 3-month therapeutic anticoagulation is recommended. Thrombolytic therapy in patients with COVID-19 is not recommended, except in those with high-risk PE with hemodynamic compromise [28]. The above recommendations are in agreement with the International Society on Thrombosis and Haemostasis guidance on the recognition and management of coagulopathy in COVID-19 [6]. Adams et al. [29] emphasized that in COVID-19 patients with significantly elevated D-dimer levels, full anticoagulation appears to provide more benefits. Full-therapeutic-dose anticoagulation is recommended for patients with increased fibrinogen (>8 g/L) or D-dimer (>3.0 μg/mL) levels. This recommendation may be supported by the fact that elevated fibrinogen levels may compromise the efficacy of heparin-based VTE prophylaxis [30]. Practical guidance on VTE prevention and treatment in patients with COVID-19 was also published by the COVID-19 Scientific Forum of the Chamber of Physicians and Dentists (in Polish: Forum Naukowe COVID-19 Naczelnej Izby Lekarskiej). These guidelines are in line with other expert recommendations. As an add-on, they include sections on VTE management at discharge and on home-based treatment. Based on current expert recommendations, an example of a thromboprophylaxis and anticoagulant treatment regimen in adult patients hospitalized due to COVID-19 is shown in Figure 1.

However, considering the differences in coagulopathy and prothrombotic potential in patients with vs. those without COVID-19, the above guidelines seem to be insufficient. Moreover, it was reported that patients with COVID-19 may develop VTE, including PE, despite heparin-based anticoagulation, including therapeutic-dose heparins [12,13]. This may necessitate updates in the guidelines on the management of this particular patient population. Barrett et al. [31] suggested that in patients with severe COVID-19, therapeutic anticoagulation with unfractionated heparin should be considered, and that antithrombin supplementation may be of utility [31]. In the setting of COVID-19 and related thromboembolic complications, the efficacy of fibrinolytic therapy becomes an important topic. Patients with VTE, including PE, were reported to have impaired fibrinolysis associated with a more compact fibrin structure [32]. Moreover, fibrin accumulation was shown to promote alveolar damage and hyaline membrane formation [33]. Maier et al. [34] assessed differences in fibrinolysis stimulated by tPA by comparing the plasma of patients with COVID-19 on prophylactic heparin therapy with that of healthy donors. The addition of tPA resulted in lower fibrinogen levels (37.9% ± 16.5% vs. 58.9% ± 18.3%, *p* = 0.0035) and a longer time to lysis (48.8 ± 16.3 min vs. 30.5 ± 15.4 min, *p* = 0.0053) in patients with COVID-19 [34]. Differences in the features of hemostatic disorders in patients with COVID-19 were also confirmed by Blasi et al. [35]. In their study, patients with COVID-19 were shown to have enhanced clot formation and reduced fibrinolytic potential despite heparin therapy. Moreover, the hypercoagulable state is more prominent in more severely ill patients [35]. These findings are in line with other studies. Hammer et al. [36] assessed whole-blood samples from COVID-19 patients to assess coagulation and fibrinolysis. COVID-19 patients showed increased resistance to lysis and a longer lysis time after stimulation with tPA, as compared with controls (maximum lysis: 3.25 ± 0.56% vs. 6.20 ± 0.89%, *p* = 0.0127; lysis time: 365.7 ± 44.6 vs. 193.2 ± 16.3 s, *p* = 0.0014).

## 7. Monitoring of Coagulation and Fibrinolysis: Thromboelastography and Rotational Thromboelastometry

Thromboelastography and rotational thromboelastometry are viscoelastic methods that provide a dynamic picture of clot formation, stabilization and lysis in whole blood. Moreover, they are used for the assessment of fibrinolysis [37,38]. These useful point-of-care tools have an advantage over conventional laboratory testing in that they can rapidly detect changes in coagulation and fibrinolysis [37]. Creel-Bulos et al. [39] assessed patients admitted to the intensive care unit, 44% of whom had impaired fibrinolysis. Pavoni et al. [40] conducted a similar study in critically ill patients with COVID-19 admitted to the intensive care unit. Patients were assessed on admission and at 5 and 10 days. In line with previous research, rotational thromboelastometry confirmed a hypercoagulable state in these patients. Nougier et al. [41] assessed patients with SARS-CoV-2 infection admitted to internal disease and intensive care units. Thromboelastography showed normal thrombin generation capacity despite heparin use. Impaired fibrinolysis was found in all patients. Interesting results about the altered clot structure in COVID-19 patients were provided by Wygrecka et al. The authors compared changes in coagulation activation during the contact phase and fibrinolysis between COVID-19 patients, acute respiratory distress syndrome influenza patients and healthy subjects. The presented data showed that the pathological events described in COVID-19 create an environment that promotes FXII activation, which, combined with high levels of fibrinogen, may contribute to the formation of a compact, lysis-resistant clot in a thrombin-dependent and -independent manner [42].

Salem et al. [43] reported a hypercoagulable state in 30.8% of critically ill patients with COVID-19 despite pharmacologic thromboprophylaxis. All these studies confirm that critically ill patients with COVID-19 are susceptible to hemostatic disorders and hypercoagulability despite antithrombotic treatment. Thromboelastography and rotational thromboelastometry may help to identify patients who may benefit from fibrinolytic therapy in terms of improved clinical status [37].

## 8. Fibrinolysis in Critically Ill Patients with COVID-19: Yes or No?

Considering that coagulopathy in patients with COVID-19 has different features that have not been fully understood so far, clinicians have made attempts to use different treatment options, including fibrinolysis. Della Bona et al. [14] reported a case series of four patients who developed PE despite heparin use, including during treatment with sodium heparin. In most cases, systemic fibrinolysis with alteplase proved effective. Other authors also reported clinical improvement in critically ill patients with COVID-19 after fibrinolytic therapy [14,44,45,46]. It is suspected that pulmonary microemboli may contribute to respiratory failure in patients with COVID-19. Therefore, tPA therapy may offer clinical benefits in these patients [45,47], including those with comorbidities [48]. In a retrospective study in critically ill patients with COVID-19, So et al. [26] administered tPA to patients with a suspicion of PE. tPA infusion was associated with an improvement in 49.1% of patients. All-cause mortality in this population was 89.5% [26].

Plasminogen may be another interesting therapeutic agent in the fight against COVID-19. In a study by Della-Morte et al. [49], patients with COVID-19 with low plasminogen levels showed a 12-fold higher risk of mortality than COVID-19 patients with normal or high plasminogen levels (odds ratio, 12.57; 95% CI, 2.46–64.0; β = 2.53; *p* = 0.002). Wu et al. [50] studied the effect of atomization inhalation with freeze-dried plasminogen in patients with moderate to severe COVID-19. They showed that additional plasminogen may be effective in treating lung lesions and hypoxemia in these patients. The authors concluded that while further studies are needed, this fibrinolytic agent may be an adequate therapeutic option in this population of patients [50].

Consideration of systemic fibrinolysis should always involve a careful risk-to-benefit assessment in terms of possible adverse events. The risk of bleeding in COVID-19 patients was reported at 4.8%, with a slightly higher risk of 7.6% in those with critical disease [33]. Moreover, the initial enthusiasm as to the continuation of fibrinolytic therapy in critically ill patients with COVID-19 was dampened by the results of a randomized clinical trial in patients receiving thrombolytic therapy. Based on preliminary findings, thrombolytic therapy does not improve oxygenation in these patients. Moreover, major bleeding was reported in 10.2% of patients within 7 days of the infusion of recombinant tPA, while concomitant anticoagulation and tPA resulted in a higher risk of bleeding (in 5 of 6 patients with major bleeding) [51]. On the other hand, coagulopathy in critically ill patients with COVID-19 has prothrombotic potential. A large, multicenter, randomized controlled trial showed that the combination of tPA bolus and heparin is safe in COVID-19 patients with severe respiratory failure. None of the patients experienced major bleeding, including intracranial bleeding, although the authors noted that this might have been due to the careful patient selection [52].

Thus, based on current knowledge, it is difficult to conclusively answer the question of whether the routine use of fibrinolysis in COVID-19 patients with a severe course and/or with coexisting pulmonary embolism in hemodynamically stable patients is warranted.

Nevertheless, the distinct features of coagulopathy in this patient population have been well described. It is possible that the point-of-care testing with thromboelastography and rotational thromboelastometry could be helpful in selecting candidates for fibrinolytic therapy. Although the general vaccination program and virus evolution have changed the dynamics of the pandemic, the large discrepancies in the current literature necessitate further research to clarify treatment recommendations for patients with severe COVID-19.

## 9. Study Limitations

The lack of adequate control groups for retrospective studies is a major limitation of this review.

## Figures and Tables

**Table 1:** Abnormalities in coagulation parameters in patients with CAC and SIC/DIC [4,6,21,22].

Parameter	CAC	SIC/DIC
Platelets ^1^	Reduced platelet count is possible although not characteristic	Decreased
D-dimer	Increased	Increased
PT ^1^	Prolonged	Prolonged
aPTT	May be longer with worsening of coagulopathy	Prolonged
Fibrinogen ^2^	Initially increased and then reduced with worsening of coagulopathy	Initially normal and then reduced with worsening of coagulopathy
Antithrombin	Reduced but rarely below the lower limit of normal	Decreased

PT, prothrombin time; aPTT, activated partial thromboplastin time; CAC, COVID-19-associated coagulopathy; SIC, sepsis-induced coagulopathy; DIC, disseminated intravascular coagulation. ^1^ ISTH experts recommend D-dimer, prothrombin time and platelet testing in each patient with COVID-19. ^2^ Fibrinogen testing may also be useful.

## Data Availability

Not applicable.
